# Diabetes and abnormal glucose regulation in the adult population of Burkina Faso: prevalence and predictors

**DOI:** 10.1186/s12889-018-5257-4

**Published:** 2018-03-13

**Authors:** Tieba Millogo, Brice W. Bicaba, Joseph Kouesyandé Soubeiga, Estelle Dabiré, Isaie Médah, Séni Kouanda

**Affiliations:** 1 Institut Africain de Santé publique (IASP), Ouagadougou, 12 BP 199 Burkina Faso; 2 Institut de recherche en sciences de la santé (IRSS), Ouagadougou 03, 03 BP 7102 Burkina Faso; 3 Ministry of Health, Ouagadougou, Burkina Faso

**Keywords:** Diabetes mellitus, Risk factors, Burkina Faso

## Abstract

**Background:**

The prevalence of diabetes mellitus (DM) is reportedly growing fast in sub-Saharan Africa. There is however a scarcity of epidemiologic data on DM in Burkina Faso. We carried out a secondary analysis of the first survey conducted in Burkina Faso on a nationally representative sample following the World Health Organization (WHO) Stepwise approach to risk factors Surveillance (STEPS) for non-communicable diseases (NCDs) with the aims of identifying the prevalence of NCDs and the prevalence of common risk factors for NCDs. We report here on the prevalence of diabetes and overall abnormal glucose regulation (AGR) and their associated risk factors.

**Methods:**

In the primary study 4800 individuals were randomly sampled using a stratified multistage clusters sampling process. We used fasting capillary whole blood glucose level to define three glucose regulation statuses using WHO’s cut-off levels: normal, diabetes and overall abnormal glucose regulation (impaired fasting glucose and diabetes). Appropriate statistical techniques for the analysis of survey data were used to identify the factors associated with diabetes and abnormal glucose regulation fitting a logistic regression model. Analyses were carried out using Stata Version 14 software.

**Results:**

The prevalence of DM and AGR were respectively 5.8% (95% CI: 5–6.7) and 9% (95% CI: 8–10.1). Significant risk factors for DM include age (OR = 1.9; *P* = 0.009 for the age group of 55–64), obesity (OR: 2.6; *P* = 0.001), former smoke (OR:2; *P* = 0.03), second-hand smoke (OR = 1.7; *P* = 0.006) and total cholesterol level (OR: 2.1; *P* = 0.024). The same predictors were also found significantly associated with AGR. In addition, having an history family diabetes was protective against AGR (OR = 0.5; *P* = 0.032).

**Conclusion:**

Diabetes is no longer a rare disease in the adult active population of Burkina Faso. Its burden is significant in both rural and urban areas. Health policies that promote healthy life style are needed to give precedence to the prevention in a context of an under-resourced country.

## Background

Diabetes mellitus (DM) is one of the major public health issue of the twenty-first century [1] bearing a heavy burden of morbidity and mortality worldwide [2–4]. It is now among the top ten leading causes of death globally [5] and the number of people affected in sub-Saharan Africa (SSA) is growing fast as compared to the other regions of the world [6]. Per World Health Organization (WHO)‘s estimates, the number of people living with diabetes will rise from 171 million in 2000 to 366 million in 2030 [7]. Every year, more than 1 million people die from diabetes and about 10 million develop disabilities and life-threatening complications [7]. Three out of four people with DM live in low-and-middle-income countries [3] and it is a contributing factor to heart diseases and stroke. The rising burden of DM is an important challenge for the health systems of SSA countries where the health care provision has been long shaped to battle against emergencies and outbreaks of infectious diseases [8]. The training of the health care workforce and the systems management of the health services in place are not always adapted to chronic conditions [9]. In the face of the emerging epidemic of DM, the World Health Assembly adopted the global strategy for integrated prevention of non-communicable diseases [10]. In 2011 the International Diabetes Federation (IDF) developed the first global plan against diabetes with a view of mobilizing and guiding initiatives against diabetes. These initiatives are based on global estimates of the disease burden including data from SSA [2, 3, 6]. Most of the key determinants of DM have also been well documented in the literature. These are: socioeconomic status, obesity, Physical inactivity and growing urbanization [11–17]. However, as an ill-prepared and a region with the highest growth rates of DM over the last decade, the situation in SSA Africa requests more attention and local and specific strategies drawn on real epidemiologic data are needed. Studies conducted in this region over the past 10 years showed important disparities between countries with reported burden of the disease ranking from very low prevalence to very high prevalence [6]. Life style related factors play an important role in the occurrence of DM. These are known to be highly context specific, making it also important to study the predictors of DM in each context. Very often Type 2 diabetes is preceded by a pre-diabetes status when it is still possible to initiate timely preventive interventions [18]. Despite the acknowledge that pre-diabetes patients are at high risk of developing DM and the benefits of risk stratification for preventives strategies [19–21], very few studies in LMIC have reported on overall abnormal glucose regulation (AGR) accounting for pre-diabetes status.

In Burkina Faso, data on DM are remarkably scarce and the available estimates were derived from data of neighbour countries (“within the same data region”) that were deemed similar enough to provide accurate estimates [2]. With the recent availability of population level data obtained with the first survey on the prevalence of major risk factors for non-communicable diseases in Burkina Faso (Stepwise approach to Surveillance of chronic diseases), we aim at (i) determining the prevalence of DM and overall AGR and (ii) identifying their associated predictors in a nationally representative sample of the adult population of Burkina Faso.

## Methods

### Study settings

Burkina Faso is a landlocked country located in the heart of the west African region. Around half of its 18 million inhabitants live below the line of poverty and the majority of the population (77.30%) live in rural areas [22]. The country faces a persistent high burden of diseases with a mix of endemic and epidemic diseases [23]. In recent years, a gradual increase in the burden of non-communicable diseases has been observed [24] along with traditional infectious diseases such as malaria and meningitis. The prevalence of DM was estimated at 3% in 2011.

### Study design and population

We carried out a cross-sectional study that consisted of a secondary analysis of the STEPS survey data. The survey was carried out on a nationally representative sample of adults from September 26 to November 18, 2013.

The study participants were adults of both sex (men and women) residing in the country during the data collection period. Were considered in the survey individuals with the following criteria: age greater or equal to 25 years and less or equal to 64 years on the day of the survey and being resident in the country for at least the last six (06) months. People with important disabilities (serious mental disorder, hearing or intellectual disability) that affect their ability to answer the survey questions were excluded from the survey. An appointment was made if the selected person in the household was absent on the day of the survey. Selected individuals who remained absent after two (02) unsuccessful visits to the households were classified as refusal.

### Sampling and sample size calculation

A stratified three-stage cluster sampling proportional to the size was used to select participants in the study. The enumeration areas (EAs) constructed from the 2006 general census of the population and housing (GCPH 2006) and updated in 2010 during the Demographic and Health Survey (DHS) in Burkina Faso serve as clusters. A full description of the EAs is available elsewhere [25]. The sample was stratified to ensure adequate representation of both rural and urban residential status. An excel spread sheet was used to draw the households to be surveyed from each selected cluster; at the third stage, the selection of individuals in households was made randomly using the Kish method [26]. In each household, one individual aged 25–64 years residing in the household was selected to participate in the survey. In the primary study the sample size calculation was based on the prevalence of hypertension as primary outcome. It was estimated that a minimum sample size of 4785 was required to measure the prevalence of hypertension with a precision of 5% with an anticipated prevalence of hypertension estimated at 29,4% and accounting for the design effect set at 1.5 and the subgroups analysis with 8 subgroups (4 age groups and 2 sex groups or rural vs urban groups). The non-response rate was anticipated to be 20%. The minimum sample size was calculated using the following formulae: $n=\left(\frac{Z_{\alpha}^2\ast P\left(1-P\right)}{e^2}\ast 1.5\ast 8\right)/0.8$. The survey was conducted on a sample of 4800 persons and 4417 were finally included in our analysis. With this sample size and diabetes prevalence estimated at 4% in the unexposed group (general population) and an assumed increase risk of 1.5 times among exposed group, our study has 86.2% statistical power.

The data collection consisted in face-to-face interviews conducted using a standardized questionnaire adapted from WHO’s STEPS surveys questionnaires. The questionnaires were implemented in Personal Digital Assistants (PDAs). Physical and biochemical measurements were carried out on the same day. Biochemical measurements comprised blood glucose level, cholesterol (total, LDL and HDL) and triglycerides levels and were conducted on fasting participants. A minimum of 8 h fasting was required and an appointment for the next day was made for participants that did not meet this criterion. Data collection was conducted by fieldworkers with medical training background (either medical student or nurses) supervised by a study medical doctor. Fieldworkers underwent a 5-days training including a field pre-test of the study instruments prior to the commencement of the real survey.

### Study variables measurements and definitions

**Fig. 1:**
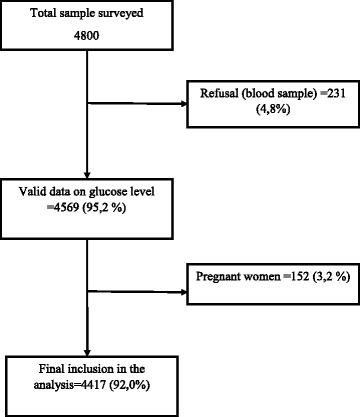
Diagram flow of the study participants. As we analysed secondary data, this figure depicts the criteria that were applied in selecting the study participants and the numbers that were affected by these criteria

The glucose level was measured using fasting capillary whole blood glucose level. All biochemical measurements were obtained using a portable device (*CardioChek P•A™ SILVER)* that performs the glucose, the cholesterol and triglycerides measurements using a whole blood sample obtained from a finger prick. This technique is analogous to the “point-of-care testing” widely used for the diagnosis and monitoring of diabetes [27, 28]. The WHO cut-offs levels for capillary whole blood glucose were used to define different glucose regulation statuses (normal, abnormal and diabetes). A blood glucose level < 5.6 mmol/l (100 mg/dl) was classified as normal glucose regulation. Participants with a blood glucose level ≥ 6.1 mmol/l (110 mg/dl) or those that reported current use of anti-diabetic treatment were defined as having diabetes. Overall AGR status was defined by either having diabetes or having a blood glucose level ≥ 5.6 mmol/l (100 mg/dl) [29]. The above cut-points for diabetes and AGR are defined for whole blood glucose measurements. Recent published cut-points are based on plasma glucose measurements and are as following: normal (≤6 mmol/l), diabetes (≥7 mmol/l) and AGR (6.1–6.9 mmol/l) [30]. Whole blood measurements can be converted into plasma levels. Adding a converting factor of 1.1 to the whole blood glucose level to derive the equivalent plasma level was found reasonable [31]. This conversion is however subject to caution as the difference between the two measurements depends on the haematocrit and likely other personal characteristics [32]. The sensitivity analysis carried out using the cut-points for plasma glucose showed no significant difference in prevalence of diabetes using both types of cut-points. The prevalence of the overall AGR was however different using both types of cut-points. We reported both results in Fig. 1. Because equating glucose levels from one measuring procedure to another is subject to uncertainties and different converting factors are found in the literature [33], we only interpreted the results based on the cut-points for whole blood measurements.

The total cholesterol level was considered high if > 2 g/l (5.2 mmol/l). Socio-demographic and behavioural factors were also recorded: aside the socio-demographic characteristics (gender, age, marital status, educational status and occupation), the questionnaire covered smoking habits, alcohol use, dietary habits, and physical activity pattern. The WHO STEPS questionnaire is a validated questionnaire [34] that have been broadly in use in several settings for years. Second-hand smoke was defined by regular exposition to smoking in one’s home or closed working place and former smoking referred to previous smokers who reported having quitted smoking.

Anthropometric measures recorded include the height, the weight and the waist. The height was measured in cm using a statometer on a subject without shoes and the weight was measured in kg using a person scale on subject in light clothing and without shoes. The Body Mass Index (BMI) was calculated as weight (kg)/height2 (m) and was used as a measure of total body obesity while the waist circumference (cm) was used as a marker of abdominal obesity. The BMI was considered normal if between 18.5 and 24.9, underweight if < 18.5, overweight if between 25 and 29.9, and obese if > 30. The waist circumference was considered: normal (less than 94 cm for men and less than 80 cm for women) or high (greater than or equal to 94 cm for men and higher than 80 cm for women).

### Statistical analysis

The dependent variables were diabetes status and abnormal glucose regulation. All analyses were performed using Stata version 14.1. All the analyses were weighted based on the probability of each participant to be selected in the sample and based on complete cases analysis. We computed the standardized prevalence using direct standardization and WHO world standard population as standard population. We first re-calculated the percentages of the WHO world standard population that would have been in each age group if it was restricted to the age span (25–64) of our population. We then derived the standardized age specific prevalence by multiplying each age specific prevalence found in our population by the corresponding population proportion in the standard population. And we finally sum up the standardized age specific prevalence to get the overall standardized prevalence.

In univariate analysis, Chi-square tests and Fisher exact test were used for categorical covariates and independent two samples t-test for continuous covariates. We fitted a multivariable logistic regression model to determine the factors associated with diabetes and abnormal glucose regulation. Robust standard errors were computed to account for the clustered nature of the data. Epidemiologic relevance was used to select variables in the multivariable analysis. Nested models were compared using the Akaike’s and Bayesian information criteria (AIC and BIC). The rule of parsimony was applied to choose the final model. Associations were considered significant if *p* ≤ 0.05.

## Results

### Background characteristics

Out of the 4800 respondents, 231 (4.8%) were excluded from the analysis because of refusal and missing data on blood glucose. Also152 women that were pregnant (3.2%) at the time of the survey were excluded from the analysis (see Fig. 1). We analysed a total of 4417 individuals (92.0%), among whom 2218 (52.7%) were female. The age group 25–34 years was the most represented (40.7%) and a vast majority of the participants had never gone to school (77.5%) and were married (72.8%). The overall prevalence of obesity was 4.9% and 849 (19.9%) had high blood pressure. Regarding family history of diabetes, 4.8% of respondents had a history of diabetes in the family. Further characteristics of the study sample and their distribution per glucose regulation status are summarized in Table 1.

**Table 1:** Background characteristics of study participants according to blood glucose regulation status

	All sample		Glucose regulation status					
			Normal		Have diabetes		Have AGR^a^	
	n	%	n	%	n	% (95% CI)	n	% (95% CI)
Age groups								
25–34 years	1943	40.7	1817	93.4	80	4.5 (3.5–5.8)	126	6.6(5.4–8.0)
35–44 years	1124	27.9	1018	91.0	69	5.8(4.4–7.5)	106	9.0(7.2–11.1)
45–54 years	819	19.4	736	89.5	50	6.9(5.0–9.5)	83	10.5(8.2–13.4)
55–65 years	531	12.0	466	85.2	43	8.7(6.2–12.2)	65	14.8(11.3–19.1)
Gender								
Male	2199	47.3	2006	90.3	128	6.3(5.1–7.7)	193	9.7(8.2–11.4)
Female	2218	52.7	2031	91.7	114	5.4(4.4–6.7)	187	8.3(7.1–9.8)
Education								
None	3418	77.5	3136	91.4	173	5.3(4.5–6.3)	282	8.6(7.5–9.8)
Primary	680	15.1	618	90.4	46	7.6(5.4–10.6)	62	9.6(7.2–12.8)
Secondary	212	5.1	187	87.4	16	7.9(4.5–13.4)	25	12.6(7.9–19.5)
Tertiary	99	2.3	88	90.4	7	7.1(3.2–15.1)	11	9.6(5.0–17.6)
Residence								
Urban	963	27.2	860	88.9	72	8.0(6.1–9.4)	103	11.1(8.9–13.9)
Rural	3454	72.8	3177	91.8	170	5.0(4.2–6.0)	277	8.1(7.2–9.3)
Marital Status								
Single	318	6.9	295	93.6	14	4.6(2.5–8.2)	23	6.4(4.0–10.1)
Married	3793	87.0	3466	91.1	215	6.0(5.1–7.0)	327	8.9(7.9–10.1)
Divorced/Widow	301	6.1	271	87.1	13	5.0(2.7–9.1)	30	12.9(8.6–18.9)
Smoking Status								
Never	2147	49.2	1981	92.6	97	4.4(3.5–5.5)	166	7.4(6.2–8.8)
Current Smoker	901	19.6	825	90.6	50	6.0(4.4–8.2)	76	9.4(7.3–12.2)
Former Smoker	141	2.8	118	83.5	15	10.6(6.0–18.1)	23	16.5(10.6–24.7)
Second Hand Smoking	1227	28.4	1112	89.4	80	7.7(6.0–10.0)	115	10.6(8.7–13.0)
History of diabetes								
No	3484	79.3	3175	90.5	199	6.2(5.3–7.2)	309	9.5(8.3–10.8)
Yes	204	4.8	190	93.8	11	4.7(2.3–9.2)	14	6.2(3.4–11.2)
Don’t know	729	15.9	672	92.7	32	4.5(3.0–6.6)	57	7.3(5.5–9.8)
HBP								
No	3568	80.1	3302	92.1	170	5.3(4.4–6.3)	266	7.9(6.9–9.1)
Yes	849	19.9	735	86.8	72	8.2(6.3–10.5)	114	13.2(10.7–16.2)
Daily frut intake								
< 5 Portions	4208	96.4	3867	91.7	221	5.4(4.6–6.3)	341	8.3(7.4–9.4)
> =5 Portions	160	3.6	127	74.5	18	16.3(9.5–26.5)	33	25.5(17.3–35.8)
Waist circumference								
Normal	3773	83.8	3474	91.5	188	5.4(4.5–6.3)	299	8.5(7.5–9.7)
Elevated	640	16.2	560	88.7	53	8.1(6.0–11.0)	80	11.3(8.9–14.4)
BMI								
18.5–25	3135	69.5	2898	92.2	149	4.8(4.0–5.8)	237	7.8(6.7–9.0)
< 18.5	498	11.9	449	88.5	27	6.5(4.1–10.2)	49	11.5(8.3–15.7)
> 25–29.9	580	13.7	517	89.9	42	7.0(5.0–9.7)	63	10.1(7.6–13.3)
> =30	192	4.9	163	83.2	23	15.1(9.5–23.0)	29	16.8(11.1–24.7)
Total cholesterol								
Normal	4291	97.1	3937	91.4	224	5.5(4.8–6.5)	354	8.6(7.6–9.6)
Elevated	124	2.9	98	76.6	18	15.5(9.3–24.7)	26	23.4(15.2–34.2)
Physical activity								
Low	648	18.1	581	89.0	49	8.2(6.0–11.2)	67	11.0(8.4–14.2)
Moderate	1045	27.5	957	91.7	58	5.8(4.3–7.8)	88	8.3(6.5–10.5)
High	2146	54.4	1958	90.7	114	5.6(4.5–6.9)	188	9.3(7.9–11.0)
Alcohol use								
Never	3036	68.7	2797	92.0	153	5.4(4.5–6.5)	239	8.0(6.9–9.3)
Every Day	291	6.6	264	88.5	17	6.0(3.6–10.0)	27	11.5(7.4–17.3)
3–6 Times per Week	322	7.2	284	89.1	24	6.4(4.1–10.0)	38	10.9(7.6–15.5)
1–2 Times per Week	388	9.0	350	90.8	24	6.1(3.7–9.8)	38	9.2(6.3–13.2)
< 1 Day Per Week	375	8.5	337	86.9	24	8.2(5.1–13.0)	38	13.1(9.1–18.3)
Standardized prevalence (using WHO world standard population)						6.1(5.4–6.9)		9.6(8.7–10.5)

^a^*AGR* Abnormal Glucose Regulation

### Prevalence of diabetes and AGR

**Fig. 2:**
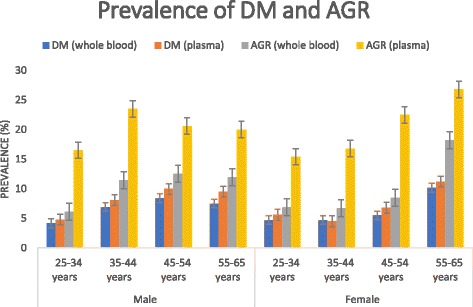
Prevalence of DM and AGR per age groups and sex. We present in this figure the prevalence of diabetes mellitus and overall abnormal glucose regulation per age groups and stratified by gender using both types of cut-points: The cut-point for whole blood glucose measurement and the cut-point for plasma glucose measurement. 95% CI intervals were added to the figures

The overall prevalence of DM in our study was 5.8% (CI_95%_: 5–6.7). The lower prevalence was noted in the Cascades region with 1.3% while the highest pertained to the Centre region with 10.6% (result not shown). The prevalence of DM was higher in urban area (8%; (CI_95%_: 6.1–10.4) as compared to rural area (5%; CI_95%_:4.2–6.0). Among all those that were classified diabetics, only 3,6% previously knew their disease status before the survey. There was no statistically significant difference between the prevalence of DM in males (6.3%; CI_95%_: 5.1–7.7) and females (5.4%; CI_95%_:4.4–6.7). The overall prevalence of AGR was 9% (CI_95%_:8.0–10.1) and there was no significant difference between males and females (9.7%; CI_95%_:8.2–11.4 vs 8.3%; CI_95%_:7.1–9.8) and between urban and rural residents (11.1%; CI_95%_:8.9–13.9 vs 8.1%; CI_95%_:7.2–9.3). The distribution of the prevalence of DM and AGR per age groups and gender is presented in Fig. 2. In both sexes the prevalence of DM and AGR roughly increases with age. However, the increase is more marked in women after 54 years. The adjusted prevalence of DM and AGR using the WHO world standard population were respectively 6.1% (CI_95%_: 5.4–6.9) and 9.6% (CI_95%_: 8.7–10.5).

### Predictors of DM and AGR

The results from the multivariate analysis are presented in Table 2.

**Table 2:** Risk factors for DM^a^ and AGR^b^ in multivariable analysis

	Diabetes						AGR					
	Univariate analysis			Mutivariable analysis			Univariate analysis			Mutivariable analysis		
	OR^c^	95% CI	*P*-value	AOR^d^	95% CI	*P*-value	OR	95% CI	*P*-value	AOR	95% CI	*P*-value
Age groups												
25–34 years	1			1.0			1			1.0		
35–44 years	1.3	0.9–1.9	0.203	1.2	0.8–1.9	0.313	1.4*	1.0–1.9	0.038	1.3	1.0–1.9	0.086
45–54 years	1.6*	1.0–2.4	0.040	1.5	1.0–2.4	0.062	1.7**	1.2–2.4	0.004	1.6*	1.1–2.3	0.011
55–65 years	2.0**	1.3–3.2	0.002	1.9**	1.2–3.0	0.009	2.5***	1.7–3.6	0.000	2.3***	1.5–3.4	0.000
Gender												
Male	1			1.0			1			1.0		
Female	0.9	0.6–1.2	0.329	0.9	0.6–1.3	0.548	0.8	0.7–1.1	0.196	0.9	0.7–1.2	0.401
History of diabetes												
NO	1			1.0			1			1.0		
YES	0.7	0.4–1.6	0.443	0.5	0.3–1.1	0.083	0.6	0.3–1.2	0.174	0.5*	0.3–0.9	0.032
Don’t know	0.7	0.5–1.1	0.135	0.8	0.5–1.2	0.263	0.8	0.5–1.1	0.109	0.8	0.6–1.1	0.211
Residence												
Urban	1			1.0			1			1.0		
Rural	0.6**	0.4–0.9	0.005	0.8	0.5–1.2	0.303	0.7*	0.5–0.9	0.020	0.8	0.6–1.2	0.349
Education												
None	1			1.0			1			1.0		
Primary	1.5	1.0–2.2	0.069	1.2	0.8–1.9	0.298	1.1	0.8–1.6	0.504	1.1	0.7–1.5	0.728
Secondary	1.5	0.8–2.8	0.188	1.0	0.5–2.1	0.957	1.5	0.9–2.6	0.119	1.2	0.7–2.2	0.517
Tertiary	1.4	0.6–3.2	0.482	0.9	0.4–2.4	0.906	1.1	0.6–2.3	0.738	0.9	0.4–2.0	0.840
BMI												
18,5–25	1			1.0			1			1.0		
< 18,5	1.4	0.8–2.3	0.229	1.4	0.8–2.4	0.214	1.5*	1.0–2.3	0.030	1.5*	1.0–2.3	0.041
> 25–29,9	1.5	1.0–2.2	0.063	1.3	0.8–2.1	0.233	1.3	0.9–1.9	0.107	1.2	0.8–1.7	0.354
> =30	3.5***	2.0–6.1	0.000	2.6***	1.5–4.7	0.001	2.4***	1.5–4.0	0.001	1.8*	1.1–3.1	0.025
Smoking Status												
Never	1			1.0			1			1.0		
Current Smoking	1.4	0.9–2.1	0.118	1.3	0.9–2.1	0.198	1.3	0.9–1.8	0.133	1.2	0.8–1.7	0.400
Former Smoking	2.6**	1.3–5.0	0.005	2.0*	1.1–3.9	0.030	2.5**	1.4–4.2	0.001	2.0*	1.1–3.3	0.013
Second Hand Smoking	1.8**	1.3–2.6	0.001	1.7**	1.2–2.4	0.006	1.5**	1.1–2.0	0.009	1.4*	1.0–1.9	0.030
Total cholesterol												
Normal	1			1.0			1			1.0		
Elevated	3.1***	1.7–5.7	0.000	2.1*	1.1–4.0	0.024	3.3***	1.9–5.6	0.000	2.6**	1.4–4.7	0.002

^a^Diabetes Mellitus

^b^Abnormal Glucose Regulation

^c^Odds ratio

^d^Adjusted OR

*p < 0.05

**p < 0.01

***p < 0.001

Our analyses identified the following as significant risk factors for diabetes mellitus:

Age, with greater age exposing to greater risk of diabetes. As compared to 25–34 years old, being in the age groups of 45–54 years and 55–64 years increased someone’s odds of having diabetes respectively 1.5 (*p* = 0.062) and 1.9 (*p* = 0.009) times. Obesity, as compared to normal, obese persons had more than twofold odds increased of having diabetes (OR = 2.6; *p* = 0.001); Former smoke and second-hand smoke increased respectively 2 times (OR = 2; *p* = 0.03) and 1.7 times (OR = 1.7; *p* = 0.006) the odds of DM as compared to never smoke.

Elevated total cholesterol level was associated with increased odds of DM (OR = 2.1; *p* = 0.024). The same predictors were also found significantly associated with AGR with the noticeable difference that persons with an history of DM in their family were 50% less likely to have an AGR (OR = 0.5; *p* = 0.032) as compared to those who didn’t have such antecedent. As compared to normal BMI, underweight persons had also a 50% odds (0R = 1.5; *p* = 0.041) increase of having an AGR.

## Discussion

Diabetes mellitus is growing fast in SSA. Our study sought to provide current and valid epidemiological estimates from Burkina Faso. The prevalence of DM and AGR were respectively 5.8% and 9% in our study. There was no significant association between both glucose regulation statuses and gender. We found a rural versus urban significant difference for the prevalence of DM and not for the prevalence of overall AGR. Smoking status (former smoke and second-hand smoke), BMI (obesity), age (greater age) and total cholesterol (high level of total cholesterol) were significantly associated with and increased odds of DM and AGR. The prevalence of DM in our findings is higher than that reported for Burkina Faso in the global estimates of diabetes by Whiting et al. [2] in 2011 (3.0%). It’s even higher than the projected level for the year 2030 in the same study (3.6%). Our prevalence is however similar to the average prevalence for the whole Africa in the same study (5.9%). Also, comparable to our result was that reported by Hilawe et al. [35] who found in a meta-analysis an average diabetes prevalence of 5.7 [95% CI 4.8 to 6.8] in sub-Saharan Africa. Previous prevalence of DM in Burkina Faso was derived from either smaller size studies or from data pertaining to neighbour countries of Burkina Faso. To the best of our knowledge, our study is the first to report on a national representative sample. We found a difference between the rural and urban areas in the burden of DM, but this difference did not hold in multivariable analysis. The prevalence of overall AGR was similar between rural and urban residents suggesting finally that the burden of the disease may not truly differs between rural and urban areas. Findings of similar prevalence in rural and urban areas were reported by Baldé N. et al. [12] in Guinea. However, these results contrast with that reported in other studies in Africa [11, 36] which showed a significantly higher prevalence in urban compared to rural area. The differences between studies may reflect true contextual differences in life styles (northern Africa vs western Africa). Albeit we saw differences in physical activity, fat intake, BMI and lipid profiles between urban and rural areas, it seems that both rural and urban residents are equally affected by the rising burden of diabetes in Burkina Faso.

### Factors associated with diabetes in Burkina Faso

As for many NCDs, ageing is a risk factor for diabetes. The 45–54 and 55–64 years age groups were more likely to have diabetes compared to the age group of 25–35 years. These results are similar to those reported in other studies conducted elsewhere [12, 24, 25].

The role of sex is however more controversial. Our study observed no relation between sex and both DM and AGR. Duboz et al. [14] in Senegal noted a risk 1.59 times higher in women (*P* < 0.05), while a meta-analysis of 36 cross-sectional studies found overall similar prevalence between both sexes (OR: 1.01, 95% CI: 0.91 to 1.11) with however, regional specificities: higher prevalence among women in southern Africa, lower prevalence among women in East and Central Africa and in low- income sub-Saharan Africa [35].

The anthropometric and metabolic factors associated with diabetes in our study are BMI and total cholesterol. The role of these factors has been broadly reported. A meta-analysis conducted in 49 developing countries [6] noted that malnourished people with overweight or obesity were more exposed to getting diabetes than those with a normal BMI. Mayega et al. [16] in Uganda, Duboz et al. [14] in Sénégal also showed that overweight / obesity was a risk factor for diabetes. Total cholesterol is also a known biomarker associated with diabetes [37]. We believe that the prevalence of poor lipid profile along with the high prevalence of overweight / obesity (18.6%) as well as eating habits will be important contributing factors to the increase in the prevalence of diabetes in the future. Former smoke and second-hand smoke were found to be significant predictors of DM and AGR in our study. The association between smoking status and DM was not confirmed in many studies. The difference may be due to these studies looking only at current and personal smoking. A smoker with fragile health status may quit smoking or urge to do so by health care workers making it difficult to find the association between current smoking and the disease. In opposite to some other studies [16–18], family history of diabetes was protective against DM and AGR in our study. The knowledge that one is at greater risk of DM may drive relatives of affected patients to have a healthier life style.

### Study limitations

We report here the results of the first nationally representative survey on the prevalence and risk factors for diabetes in Burkina Faso. The study is however entitled to some limitations. The first limitation stems from the cross-sectional nature of the data that limits the possibility of deriving causal inferences. Glucose measurements were taken at only one occasion and could not be repeated as requested for the diagnosis of diabetes in the gold standards [38] and the device used performs whole blood glucose measurement instead of plasma glucose widely used in recent studies. The measurement of most exposures was based on the recall of respondents and some well-known risk factors for diabetes were not included in the study because data on these variables have not been collected during the STEPS survey. Part of such variables is the socio-economic status.

## Conclusion

We report on the burden of diabetes and AGR and their associated risk factors in a nationally representative sample of the adult population in Burkina Faso. Our data showed an important burden of diabetes in Burkina Faso with urban and rural settings equally affected. Many risk factors identified in this study are modifiable. Appropriate health policies that promote healthy life style are thus needed to curb this disease which will be one of the main concerns in public health over the next 50 years worldwide.

## Abbreviations

BMI: Body mass index
CI: Confidence interval
EA: Enumeration area.
HBP: High Blood Pressure
HIV: Human Immunodeficiency Virus
NCD: Non-Communicable Diseases
PDA: Personal Digital Assistant
RGPH: General Census of Population and Housing
WHO: World Health Organization
